# Defining the Problem History Heterogeneity in Patients with Alzheimer’s Disease: A Data‐Driven Approach

**DOI:** 10.1002/alz.087396

**Published:** 2025-01-03

**Authors:** Clayton Mansel, Ryan A Townley, Robyn A Honea, Mihaela E Sardiu, Olivia J Veatch

**Affiliations:** ^1^ University of Kansas Medical Center, Kansas City, KS USA; ^2^ University of Kansas Alzheimer’s Disease Research Center, Fairway, KS USA

## Abstract

**Background:**

The medical and social history of patients with Alzheimer’s Disease is heterogeneous with many interacting genetic and environmental factors contributing to an individual’s risk. Moreover, a maternal family history (mFH) is a key risk factor for AD—raising the risk for disease onset by as much as nine times. However, a proportion of individuals do not have a complete knowledge of their family history. Determining which problem history profiles are common among individuals with a mFH of AD may help identify these high‐risk patients. Mitochondria are inherited matrilineally and certain haplogroups have been linked to an increased risk for AD. Subgrouping AD patients based on problem and family history using novel methods such as unsupervised machine learning (ML) may provide key insights into the pathogenesis of AD and could augment future clinical trial enrollment.

**Method:**

In this cross‐sectional study of longitudinal clinical data from the National Alzheimer’s Disease Coordinating Centers (NACC), we used agglomerative hierarchical clustering on principle components to group subjects based on self‐reported medical history and substance use history. A total of 2,754 individuals who did not have AD at their initial visit but later developed AD were included in the final analysis. Secondarily, we tested whether these clusters were associated with a mFH of AD, mitochondrial haplogroup, *APOE* genotype, and various demographic factors.

**Result:**

The final dataset had a mean age of 76±8.2 years and was 44.9% (n = 1,237) male, 84.9% (n = 2,337) white, 94.4% (n = 2,601) non‐Hispanic, and had 15.7±6.0 years of education. Three distinct problem history clusters were identified: minimal problem history (n = 1,380), substance use history (n = 1,038), and cardiovascular problem history (n = 336). The cardiovascular problem history cluster had a significantly lower proportion of subjects with a mFH of AD and an E4/E4 *APOE* genotype.

**Conclusion:**

In this study, three distinct problem history subgroups were identified. The cardiovascular problem history cluster was not associated with an E4/E4 *APOE* genotype or a maternal family history of AD, indicating that this subgroup of patients may require further study to determine how to best stratify their risk of AD.

